# Longitudinal growth of children born with gastroschisis or omphalocele

**DOI:** 10.1007/s00431-023-05217-4

**Published:** 2023-10-11

**Authors:** Asta Tauriainen, Samuli Harju, Arimatias Raitio, Anna Hyvärinen, Tuomas Tauriainen, Ilkka Helenius, Kari Vanamo, Antti Saari, Ulla Sankilampi

**Affiliations:** 1 Department of Pediatric Surgery, University of Eastern Finland and Kuopio University Hospital, Puijonlaaksontie 2, 70210 Kuopio, Finland; 2 Department of Pediatric Surgery, University of Turku and Turku University Hospital, Turku, Finland; 3 Department of Pediatrics, Kuopio University Hospital and University of Eastern Finland, Kuopio, Finland; 4 Faculty of Medicine and Health Technology, University of Tampere and Tampere University Hospital, Tampere, Finland; 5 Department of Surgery, Mehiläinen Länsi-Pohja Oy, Kemi, Finland; 6 Department of Pediatric Surgery, Oulu University Hospital and University of Oulu, Oulu, Finland; 7 Department of Cardiac Surgery, University of Turku and Turku University Hospital, Turku, Finland; 8 Department of Orthopedics and Traumatology, University of Helsinki and Helsinki University Hospital, Helsinki, Finland

**Keywords:** Gastroschisis, Omphalocele, Growth, Weight, Height

## Abstract

Normal childhood growth is an indicator of good health, but data addressing the growth of children born with abdominal wall defects (AWDs) are limited. The detailed growth phenotypes of children born with gastroschisis or omphalocele are described and compared to peers without AWDs from birth to adolescence. Data from 183 gastroschisis and 144 omphalocele patients born between 1993 and 2017 were gathered from Finnish nationwide registers and electronic health records. Weight (*n* = 3033), length/height (*n* = 2034), weight-for-length (0–24 months, *n* = 909), and body mass index measures (2–15 years, *n* = 423) were converted into sex- and age-specific *Z*-scores. Linear mixed models were used for comparisons. Intrauterine growth failure was common in infants with gastroschisis. Birth weight *Z*-scores in girls and boys were − 1.2 (0.2) and − 1.3 (0.2) and length *Z*-scores − 0.7 (0.2) and − 1.0 (0.2), respectively (*p* < 0.001 for all comparisons to infants without AWDs). During early infancy, growth failure increased in infants with gastroschisis, and thereafter, catch-up growth was prominent and faster in girls than in boys. Gastroschisis children gained weight and reached their peers’ weights permanently at 5 to 10 years. By 15 years or older, 30% of gastroschisis patients were overweight. Infants with omphalocele were born with a normal birth size but grew shorter and weighing less than the reference population until the teen-age years.

*Conclusion*: Children with gastroschisis and omphalocele have distinct growth patterns from fetal life onwards. These growth trajectories may also provide some opportunities to modulate adult health.

**Table:** 

**What is Known:** *• Intrauterine and postnatal growth failure can be seen frequently in gastroschisis and they often show significant catch-up growth later in infancy. It is assumed that part of the children with gastroschisis will become overweight during later childhood.*	
**What is New:** * • The longitudinal growth of girls and boys with gastroschisis or omphalocele is described separately until the teenage years. The risk of gaining excessive weight in puberty was confirmed in girls with gastroschisis.*

**Supplementary Information:**

The online version contains supplementary material available at 10.1007/s00431-023-05217-4.

## Introduction

A defect in the abdominal wall allows the intestines to protrude outside the abdominal cavity; in gastroschisis, the intestines are freely prolapsed in the amniotic fluid, whereas in omphalocele, the intestines are protruded into the umbilical cord. The prevalence of gastroschisis and omphalocele was recently reported as 1.73 and 1.96 in 10,000 live births in Finland, respectively [1, 2]. In other studies, prevalence of gastroschisis as high as 4.9 in 10,000 live births has been observed [3]. Gastroschisis usually presents as an isolated anomaly, whereas omphalocele is often complicated with other anomalies, including karyotype aberrations [1, 2]. At present, the AWDs are usually diagnosed antenatally, and neonatal intensive care, including surgery and nutritional support, has improved survival. Although contemporary neonatal survival rates are greater than 90% [2, 4, 5], gastroschisis is associated with significant morbidity, including gastrointestinal dysfunction, need for parenteral nutrition, sepsis, and prolonged hospitalization [6, 7]. In gastroschisis, the long-term survival after infancy has not been thoroughly investigated. However, it has been reported that the majority of deaths occurs within the first 2 years of life [8]. Survival rates of omphalocele are highly dependent on the presence of associated malformations and vary between 80 and 88%. Chromosomal defects decrease the survival rate to as low as 17% [1].

Normal childhood growth is generally regarded as a surrogate of good health that extends to adult life. The anomaly itself, its associated early-life morbidity, possible associated conditions, and type of care may alter the growth of children born with an AWD. Very little data on longitudinal growth of children born with AWDs is available, especially after infancy. In the present study, we describe the growth of 327 children with gastroschisis or omphalocele in detail from birth to the age of 24 months and thereafter throughout childhood until adolescence in comparison to the reference population.

## Materials and methods

### Patients

All live born children with gastroschisis or omphalocele in Finland between January 1, 1993, and December 31, 2017, were identified in three registers maintained by the Finnish Institute for Health and Welfare (THL). The Medical Birth Register (MBR) includes data on all births and diagnoses of newborns, the Finnish Register of Congenital Malformations (FRM) contains data on fetuses and infants with at least one major congenital anomaly, and the Finnish Hospital Discharge Register (FHDR) includes data on hospital admissions. These registers were cross-checked for diagnoses of gastroschisis or omphalocele based on the International Classification of Diseases (ICD). ICD-9 was used to find codes 756.73 and 756.72 for the year 1993 and ICD-10 for codes Q79.3 and Q79.2 for 1994 to 2017. Altogether, 288 children with gastroschisis and 299 children with omphalocele were born alive in Finland between 1993 and 2017.

All children in Finland are provided with a free-of-charge growth-monitoring program from birth to adolescence at specific ages. The program includes over 20 visits at well-baby clinics or school health care facilities. Virtually, all children in Finland participate in the program [9]. Growth data are registered in various electronic health care systems during the growth-monitoring visits in primary care centers and at hospital visits. For the present study, longitudinal growth data of 183 children (54.1% boys) with gastroschisis and 144 children (57.6% boys) with omphalocele could be obtained from the hospital records and primary care growth-monitoring systems of four university hospitals (Kuopio, Turku, Tampere, and Oulu University Hospitals) and from the primary care records of Espoo city. These patients represent 63.5% of all children born with gastroschisis and 48.2% of children born with omphalocele in Finland between 1993 and 2017. The rest of the patients from non-participating hospitals were not included in the study. This resulted in a non-nationwide study design. Within the included patient population, there was variable number of measurements, since all available growth measurements were collected. Altogether, the 183 gastroschisis patients had 1215 height and 1677 weight measurements (a mean of 6.6 [4.9] height measurements and 9.6 [8.0] weight measurements per child) and 144 omphalocele patients had 819 height and 1356 weight measurements (mean height measurements 6.2 [4.7] and mean weight measurements 8.9 [7.7], respectively]. Background characteristics of the study population were gathered from the MBR (Table 1).

**Table 1:** Characteristics of the study population

	Gastroschisis			Omphalocele		
	Girls *n* = 84	Boys *n* = 99	*p*-value	Girls *n* = 61	Boys *n* = 83	*p*-value
Gestational age at birth (weeks)	36.9 ± 1.9 [29.3 − 41.3]	37.2 ± 1.6 [33.3 − 40.9]	0.303	37.9 ± 2.8 [27.7 − 42.3]	38.1 ± 2.8 [28.6 − 42.0]	0.686
Preterm birth < 37 gestation weeks	41 (48.8)	45 (45.5)	0.650	13 (21.3)	19 (22.9)	0.822
Birth weight (g)	2560 ± 620 [1000 − 4600]	2680 ± 550 [1430 − 4240]	0.172	3190 ± 750 [930 − 5010]	3250 ± 830 [1150 − 5170]	0.040
Birth weight *Z*-score	− 1.04 ± 1.30 [− 3.73 − 3.06]	− 1.15 ± 1.27 [− 3.66 − 3.17]	0.579	0.20 ± 1.65 [− 2.52 − 5.46]	− 0.07 ± 1.69 [− 4.08 − 4.72]	0.551
Birth length (cm)	46.4 ± 3.5 [38.5 − 55.0]	46.9 ± 2.9 [39.0 − 54.0]	0.436	48.8 ± 3.6 [35.0 − 55.5]	49.0 ± 3.6 [37.0 − 55.0]	0.668
Birth length *Z*-score	− 0.54 ± 1.56 [− 3.68 − 3.89]	− 1.02 ± 1.50 [− 4.11 − 3.46]	0.049	0.34 ± 1.58 [− 2.27 − 3.89]	− 0.12 ± 1.46 [− 3.07 − 3.64]	0.186
SGA	16 (19.0)	23 (23.2)	0.491	4 (6.6)	7 (8.4)	0.760
LGA	4 (4.8)	2 (2.0)	0.415	7 (11.5)	10 (12.0)	0.916
**Maternal characteristics**						
Maternal age (years)	24.6 ± 3.9 [17.4 − 35.4]	24.9 ± 4.7 [16.0 − 35.7]	0.797	30.6 ± 6.1 [19.1 − 42.0]	29.9 ± 6.0 [18.7 − 43.1]	0.514
Antenatal steroid treatment	12 (14.3)	8 (8.1)	0.180	4 (6.6)	7 (8.4)	0.760
Diabetes^a^	5 (6.0)	6 (6.1)	0.976	10 (16.4)	14 (16.9)	0.940
Smoking after first trimester	18 (21.4)	18 (18.2)	0.612	10 (16.4)	13 (15.7)	0.384
Twins	4 (4.8)	2 (2.0)	0.415	2 (3.3)	3 (3.6)	1.000
Nullipara	62 (73.8)	70 (70.7)	0.641	31 (50.8)	40 (48.2)	0.755
Vaginal birth	28 (33.3)	44 (44.4)	0.125	34 (55.7)	38 (45.8)	0.238

Categorical variables are presented as counts and percentages. Continuous variables are presented as mean and standard deviation. Range is shown in brackets

*SGA *small for gestational age, *LGA *large for gestational age, *N/A *not available

^a^Before or during pregnancy

The study was carried out according to the Finnish National and European Union guidelines. The institutional review boards of the participating university hospitals in Kuopio, Oulu, Tampere, and Turku in addition to the city of Espoo and the Finnish Institution for Health and Welfare approved the present study (THL/206/5.05.00/2017, THL/1010/5.05.00/2018).

### Growth data and statistical analyses

Potentially erroneous measurements, typing errors, and duplicated recordings in growth data were evaluated and corrected or excluded. Height and weight measurements were transformed into *Z*-scores according to the current population-based growth references based on the growth data of 73,659 healthy Finnish children born between 1983 and 2008 aged 0–20 years [10], in addition to population-based birth size reference of 753,036 infants born between 1996 and 2008 [11]. These children formed the reference population for this study. The growth outcomes included weight *Z*-score, length/height *Z*-score, and two parameters depicting body proportions, weight-for-length *Z*-score (in infants 0–24 months), and body mass index *Z*-score ([BMI] weight/height^2^). Small for gestational age (SGA) was defined as birth weight below two standard deviations (SD) and large for gestational age (LGA) as birth weight over two SD [12]. Overweight was defined as BMI *Z*-score > 85^th^ percentile.

Statistical analyses were performed with SPSS software (version 25.0, IBM Corporation, New York, USA). The normality of variables was evaluated using the Kolmogorov-Smirnov test. Categorical variables were reported as counts and percentages. Continuous variables are reported as means, standard deviations, and standard errors with *Z*-score estimates. Chi-squared, Fisher’s exact, and the Mann-Whitney *U* tests were used for univariate analyses.

Linear mixed model analyses were used to evaluate the growth of children with omphalocele or gastroschisis by time or in comparison to the healthy child population [10, 11]. The longitudinal growth data were divided into time slots around the age-specific measurement points as depicted in Supplementary table. Only one height and weight measurement per child was included in each time slot. In cases with multiple measurements, the one closest to the median of the time slot was selected for analyses. For the assessment of growth in infancy (0–24 months from term-equivalent age [TEA], namely, until corrected age of 24 months), the measurement points were classified into TEA, 1, 2, 3, 6, 9, 12, 18, and 24 months of corrected age. For the childhood growth follow-up (2–15 years or older), the several measurement points were included that were in line with the nationwide growth-monitoring age points (2, 3, 4, 5–6, 7–10, 11–14, and 15 years or over). In patients aged ≥ 15 years (range 15–21 years), the measurement closest to the age of 15 years was selected. In the age cohort ≥ 15 years, the mean age during measurement was 15.8 (1.5) years in gastroschisis and 16.7 (2.5) years in omphalocele patients. The mixed model analysis provided *Z*-score estimates and standard errors (SE) for all time slots and considered repeated observations of the same individual. These estimates were used in comparisons between time slots or to a reference population. All statistical tests were performed as two-tailed, and a *p*-value < 0.05 represented statistical significance.

## Results

### Growth of children born with gastroschisis

**Fig. 1:**
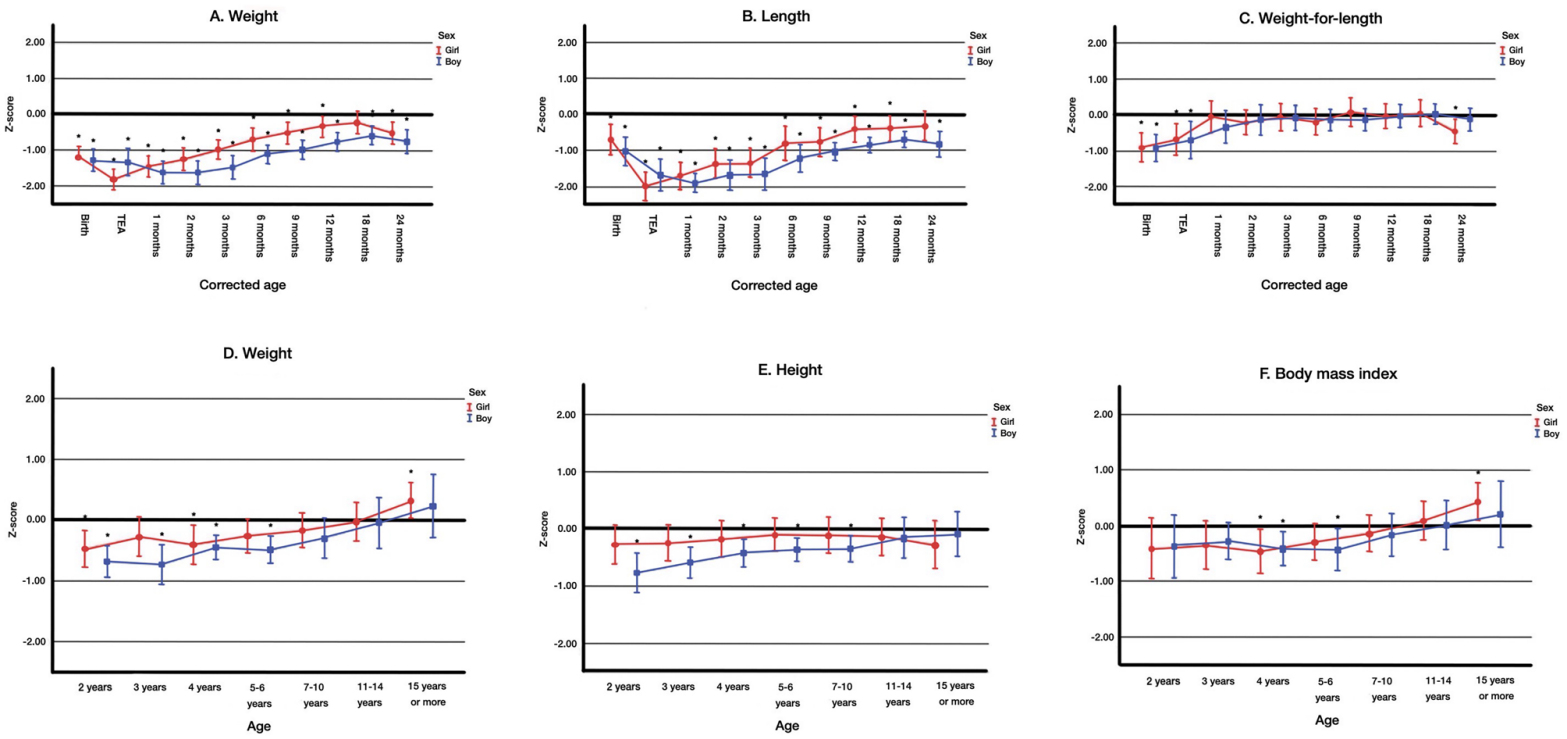
Longitudinal growth from birth to the corrected age of 24 months (**A**–**C**) and from 2 to 15 years or over (**D**–**F**) in children born with gastroschisis. Means and 95% confidence intervals (whiskers) are shown for girls (circles) and boys (squares). The zero line indicates the reference population average. Corrected age signifies the age from the term-equivalent age. Body mass index (BMI) was available only from two years onwards. * Statistically significant difference compared to the reference population (*Z*-score = 0), in the mixed model analysis (*P* < 0.05). TEA, term equivalent age

In gastroschisis, intrauterine growth failure was followed by a pronounced postnatal growth failure and a prominent catch-up growth was present in infancy. Altogether, 47% (*n* = 86) of gastroschisis patients were born preterm before the 37^th^ gestational week, and 61% of them were born by either planned or emergency C section (Table 1). The mean maternal age was 25 (4.4) years, 73% were nulliparous, 6% were diabetics, and 20% were smoking after the first trimester. At birth, infants with gastroschisis were significantly lighter and shorter than the newborns without AWDs, and 21% of them were SGA. The mean birth weight *Z*-score and SE in girls was − 1.2 (0.2) and in boys − 1.3 (0.2) (Fig. 1A) in addition to the mean birth length *Z*-scores in girls and boys were − 0.7 (0.2) and − 1.0 (0.2), respectively (*p* < 0.001 for all comparisons with the reference population) (Fig. 1B).

Postnatally, the growth failure in infants with gastroschisis increased during the first weeks or months of life (Fig. 1A–C) and the subsequent catch-up growth was slower in boys than in girls. In girls, the weight and length *Z*-scores declined from birth until TEA to − 1.8 (0.2; *p* < 0.001) and to − 2.0 (0.2; *p* < 0.001) in comparison to birth measures as shown in Fig. 1A, B, respectively. After TEA, the weight *Z*-score in girls increased faster than length *Z*-score, reaching the birth weight *Z*-score at the corrected age of 1 month (cM1) − 1.4 (0.1; *p* = 0.07), followed by the length *Z*-score at cM6 − 0.8 (0.2; *p* = 0.67). Thereafter, the weight and length *Z*-scores in girls continued to increase and reached girls without AWDs at cM18 (weight; *p* = 0.094) and cM24 (length, *p* = 0.955). In boys with gastroschisis, the weight *Z*-score estimates decreased from birth to − 1.6 (0.2) at cM2 (*p* = 0.025). In boys, the weight and length *Z*-scores reached the birth levels at the age of cM6 (*p* = 0.267 and *p* = 0.251, respectively). The boys remained lighter and shorter than boys without AWDs still at cM24 (weight *Z*-score of − 0.7 [0.2] and height *Z*-score of − 0.8 [0.2]; *p* < 0.001).

From 2 years onwards, the height *Z*-score of girls did not differ significantly from that of the girls without AWDs. The height *Z*-score of boys reached the reference population at the age of 11 to 14 years (Fig. 1E). The weight *Z*-scores in girls were significantly lower than in the reference population at the ages of two and four years (*p* = 0.002 and *p* = 0.013, respectively) and caught up to their peers permanently at the ages of 5 to 6 years. The boys were significantly lighter than the boys without AWDs until the ages of 7 to 10 years (*p* = 0.058) as shown in Fig. 1D, F. Both in girls and boys with gastroschisis, the weight in addition to BMI *Z*-score started to increase linearly from 7 to 10 years onwards during the pubertal years. At the age of 15 years or older (mean 16 years), the weight and BMI *Z*-scores in girls were significantly higher than in girls without AWDs (*p* = 0.030 and *p* = 0.011), whereas in boys, the difference was not statistically significant (*p* = 0.757).

### Growth of children born with omphalocele

**Fig. 2:**
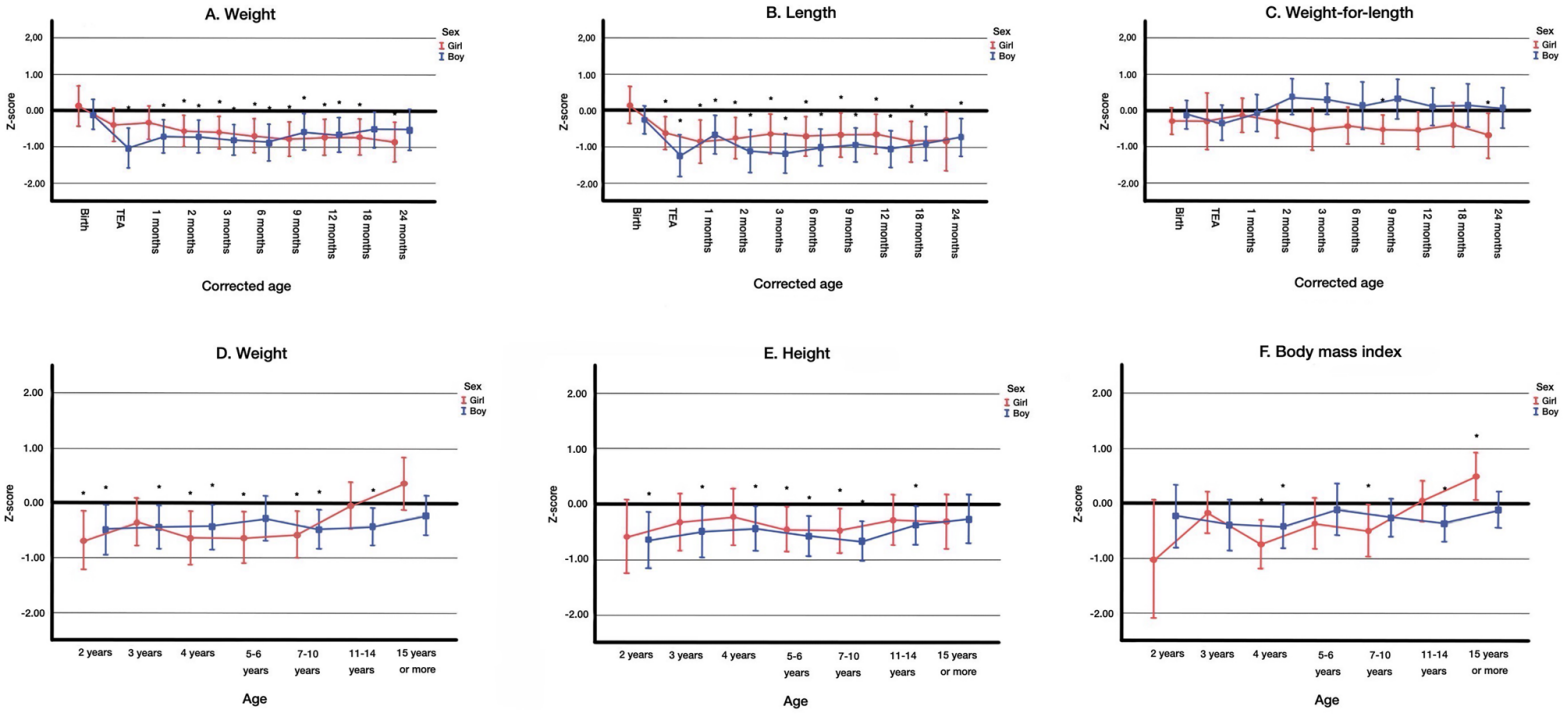
Longitudinal growth from birth to the corrected age of 24 months (**A**–**C**) and from 2 to 15 years or older (**D**–**F**) in children born with omphalocele. Means and 95% confidence intervals (whiskers) are shown for girls (circles) and boys (squares). The zero line indicates the reference population average. Corrected age signifies the age from term-equivalent age. BMI was available only from two years onwards. * Statistically significant difference compared to the reference population (*Z*-score = 0), in the mixed model analysis (*P* < 0.05). TEA, term equivalent age

Only 22% (*n* = 32) of omphalocele patients were born preterm in comparison to 47% (*n* = 86) of gastroschisis patients (*p* < 0.001) as shown in Table 1. The rate of planned or emergency C section in omphalocele was 50%. The mothers of omphalocele patients were significantly older than the mothers of gastroschisis children (30 [6.0] years vs. 25 [4.4] years, *p* < 0.001]). They were also more often multiparous 51% vs. 27% (*p* < 0.001) and diabetics 17% vs. 6% (*p* = 0.002), respectively. There was no significant difference in smoking after the first trimester between mothers of omphalocele and gastroschisis patients (16% vs. 20%, *p* = 0.284). The birth weight *Z*-scores in girls and boys with omphalocele were 0.1 (0.3) and − 0.1 (0.2) and birth length *Z*-scores 0.2 (0.3) and − 0.3 (0.2), respectively, and they did not differ significantly from the infants without AWDs (Fig. 2 A, B). Only 7.6% of omphalocele infants were born SGA. On the other hand, LGA was more common among omphalocele than gastroschisis patients (12% vs. 3.3%; *p* = 0.003).

Infants with omphalocele did not show a similar growth failure as infants with gastroschisis. However, a slow decrease both in weight and length *Z*-scores was observed in these infants during infancy. The weight *Z*-scores in girls and boys decreased from birth to cM1 − 0.3 (0.2); *p* = 0.023 and − 0.7 (0.2); *p* = 0.002 in comparison to birth size, respectively. The weight *Z*-score differed significantly from the infants without AWDs between TEA and 12 months in boys and between 2 and 18 months in girls (Fig. 2A, B). The average weight-for-length *Z*-score of infants with omphalocele was close to that of infants without AWDs from birth onwards (Fig. 2C).

The boys and girls with omphalocele were significantly shorter than infants without AWDs between TEA and 24 months and TEA-18 months, respectively (Fig. 2A, B). The same trend in the height *Z*-score continued throughout childhood, and boys with omphalocele were on average shorter than their peers until 15 years or older. The girls with omphalocele were shorter than their peers only at the ages of 2 and 5–10 years (Fig. 2E). The weight *Z*-score was significantly smaller than the reference population at the ages of 2 to 4 and 7 to 14 years in boys in addition to at the ages of 2 and 4 to 10 years in girls (Fig. 2D). By the age of 15 years, the BMI *Z*-score of girls was significantly higher than in girls without AWDs (0.5 [0.2]; *p* = 0.018). In boys (in contrast to girls) with omphalocele, similar pubertal weight gain was not seen.

## Discussion

To the best of our knowledge, no previous studies with such a long follow-up and detailed growth characterization in a large cohort of gastroschisis and omphalocele patients have been published. For both gastroschisis and omphalocele patients, distinct growth patterns could be depicted. In infants with gastroschisis, intrauterine growth failure was followed by initial postnatal growth failure and thereafter a prominent catch-up growth in infancy and average childhood growth. The same pattern was not seen in omphalocele patients who were large at birth but then grew to be slightly lighter and shorter than the reference population, probably reflecting their genetic potential and underlying conditions associated to omphalocele. In both gastroschisis and omphalocele patients, a tendency toward weight gain and increasing BMI was observed during the teenage years. The observed growth trajectories probably reflect the general health of these patients during childhood.

Our results of the intrauterine growth failure in gastroschisis patients are in line with previous observations [13–16]. Payne et al. [17] showed that birth weights of 179 gastroschisis infants born on average at 36^th^ gestation weeks was 350 g less (2400 g) than that in matched controls, and their birth lengths were an average of 2.7 cm shorter. The proportion of SGA children in our cohort was 21%, which is slightly less than in the previous cohorts (33–52%) [13–16]. This difference might reflect the quality of care at the maternity health clinic or general maternal health in the population. The pathophysiological mechanism behind the intrauterine growth failure in gastroschisis patients remains obscure.

Postnatal growth failure during the initial hospitalization period in gastroschisis has been previously observed in small study cohorts with short follow-up times. Hong et al. [18] reported significant decreases in weight *Z*-scores from birth (− 0.7) to discharge (− 1.1) in a cohort of 60 infants. Strobel et al. [19] followed 90 infants until the age of 30 days, and in 28% and 42% of them, weight and height growth failure was observed, respectively. Postnatal growth failure has been demonstrated also in three other smaller cohorts [20–22]. In our cohort, boys showed a prolonged growth period failure and delayed catch-up growth in comparison to girls. This phenomenon has not been described earlier. According to the literature, risk factors associated with poor growth in infancy among gastroschisis patients are prematurity, SGA [13, 20, 23], and central venous line infections [18].

Children born with gastroschisis often show significant catch-up growth later in infancy, which is especially seen in cases with simple gastroschisis, namely, those without bowel atresia, perforation, necrosis, or volvulus [6, 22, 24]. Giudici and colleagues [25] reported the growth of 62 gastroschisis patients, and 17 of them were followed until the age of 6 years in Argentina. Most patients grew normally from 1 to 6 years of age. Furthermore, the patients who presented with abnormal growth at 1 year of age caught up with growth and presented with normal parameters by the age of 6 years. Moreover, in a study by Hall et al. [21] that examined 61 gastroschisis infants, the catch-up growth was seen between the ages of 3 and 6 months. Minutillo et al. [26] collected growth data from 117 gastroschisis infants in Australia; these infants were found to have suboptimal weight gain during the first year of life. In the present study, more pronounced catch-up growth during infancy was seen in girls than in boys.

Data on longitudinal growth of children born with gastroschisis after infancy are scarce [24, 25]. Harris et al. [24] described weight and height of 42 children at the mean age of 9 years (range 5–17 years). Their results indicated that the *Z*-scores associated with weight and height increased significantly from birth. Ten children (24%) in their cohort were overweight at the time of follow-up. This result is in line with the present study with around 30% of girls and boys with gastroschisis being overweight (> 85^th^ percentile) at the ages of 14 to 16 years. These findings indicate that the risk of gaining excessive weight in puberty may be a true finding. This weight gain may be related to early life undernutrition and rapid catch-up growth in infancy in the gastroschisis patients, analogous to otherwise healthy infants born SGA [27]. SGA adults who gained weight rapidly in infancy show an increased risk for adult life morbidity [12]. Thus, in gastroschisis patients, the weight gain in puberty observed in this study may be an indicator of life-long metabolic risks. Previously, children with AWD have been recommended a high calorie diet; however, this practice should be re-evaluated. Follow-up studies revealing adult health and weight data of gastroschisis patients are needed.

In contrast to gastroschisis patients, those with omphalocele were similar or larger size at birth than infants without AWDs of the same gestational age, and the proportion of LGA infants was larger than among this population. The mean birth weight of 3200 g in the omphalocele infants is in line with previous studies [28, 29]. One of the most common syndromes associated with omphalocele is the Beckwith-Wiedemann syndrome (BWS), which can be seen in around 25% of omphalocele patients [28] and which can cause LGA based on hyperinsulinism.

Previous research on long-term outcome in children with omphalocele has focused mainly on motor and neurological development, but not on growth [29–31]. Hijkoop et al. [32] measured 42 omphalocele patients at the age of 24 months, and showed that the mean weight and length *Z*-scores were lower than in their reference population (− 0.9 and − 0.6, respectively). This observation is in line with the results of the present study. The heterogeneity of associated anomalies or syndromes with omphalocele patients results in a wide variety of morbidity, explaining the longitudinal growth pattern below the population average. However, a tendency toward weight gain was seen also in omphalocele patients since over 25% of them were overweight at adolescence. The growth pattern could be different in isolated omphalocele. In our study, the full genetic information of omphalocele children was not available.

The major strength of the present study was the large cohort of patients, representing over 50% of all children born with AWDs in Finland between 1993 and 2017. Furthermore, the patients’ good-quality growth data was abundant. We believe that this first longitudinal growth study addressing both gastroschisis and omphalocele patients is reliable and generalizable also to other environments with similar diagnoses. In Finland, the health care system and patient follow-up is of high quality; however, the growth patterns in patients with AWDs might be different in developing countries. The limitation is the lack of data on the initial hospitalization, nutritional status, and number of surgical operations in addition to the lack of detailed data on the associated anomalies and conditions, for example, BWS or chromosomal abnormalities. Also, adult height and BMI trajectories from adolescence to adulthood remain to be studied.

## Conclusions

Children with gastroschisis and omphalocele have some distinct growth pattern features starting in fetal life onwards. Children with gastroschisis suffer from pre- and postnatal growth failure, but after a long catch-up period, reach eventually the average adult height of the population. In contrast, children with omphalocele grow well or even excessively prenatally, but finally become slightly shorter adults than the average. Importantly, in both conditions, there might be a risk for excessive weight gain after puberty. These growth trajectories reflect their general health and may also provide some opportunities to affect adult-life metabolism. Counselling parents and patients about the longitudinal growth patterns is therefore necessary. During postnatal care, active efforts to prevent growth failure by providing adequate nutrition, and in childhood, facilitating a healthy lifestyle to prevent excessive weight gain may be beneficial for better metabolic health during adult life.

### Supplementary Information

Below is the link to the electronic supplementary material.

Supplementary file1 (DOCX 15 KB)

## Abbreviations

AWD: Abdominal wall defect
BMI: Body mass index
BWS: Beckwith-Wiedemann syndrome
cMx: Corrected age of *x* months
FHDR: Finnish Hospital Discharge Register
FRM: Finnish Register of Congenital Malformations
ICD: International Classification of Diseases
LGA: Large for gestational age
MBR: The Medical Birth Register
SD: Standard deviation
SE: Standard error
SGA: Small for gestational age
TEA: Term-equivalent age
THL: Finnish Institute for Health and Welfare
