# Supplementary material for: Longitudinal growth of children born with gastroschisis or omphalocele
Source: Eur J Pediatr. 2023 Oct 11;182(12):5615–23. doi: 10.1007/s00431-023-05217-4 (PMC10746581; doi:10.1007/s00431-023-05217-4)
Supplement: Supplementary file 1 — Supplementary file1 (DOCX 15 KB) [file 431_2023_5217_MOESM1_ESM.docx]

Supplementary table. The criteria for the selection of measurement points for growth assessment in infancy and in the childhood until adolescence.

|  | Age period | Number of measurements |
| --- | --- | --- |
| Growth in infancy, corrected age* |  |  |
| Birth | The measurements at the day of birth | N=322 |
| Term-equivalent age | −0.49–0.49 months, excluding birth measurement | N=124 |
| 1 month | 0.50–1.49 months | N=165 |
| 2 months | 1.50–2.49 months | N=126 |
| 3 months | 2.50–4.49 months | N=203 |
| 6 months | 4.50–7.49 months | N=249 |
| 9 months | 7.50–10.49 months | N=163 |
| 12 months | 10.50–14.99 months | N=148 |
| 18 months | 15.00–20.99 months | N=137 |
| 24 months | 21.00–26.99 months | N=122 |
| Childhood growth, age |  |  |
| 2 years | 18.00–29.99 months | N=215 |
| 3 years | 30.00–41.99, months | N=142 |
| 4 years | 42.00–53.99 months | N=117 |
| 5-6 years | 54.00–77.99 months | N=210 |
| 7-10 years | 78.00–125.99 months | N=333 |
| 11-14 years | 126.00–173.99 months | N=251 |
| 15 years or more | ≥174.00 months | N=186 |

* Corrected age, i.e time calculated from the term-equivalent age; Only one height and weight measurement per child was included in each age slot. In cases with multiple measurements, the one closest to the median of the age slot was selected for statistical analyses.
